# Fruit and Vegetable Prescriptions for Pediatric Patients Living in Flint, Michigan: A Cross-Sectional Study of Food Security and Dietary Patterns at Baseline

**DOI:** 10.3390/nu11061423

**Published:** 2019-06-25

**Authors:** Amy Saxe-Custack, Jenny LaChance, Mona Hanna-Attisha, Tiffany Ceja

**Affiliations:** 1 Department of Food Science and Human Nutrition, Division of Public Health, Michigan State University–Hurley Children’s Hospital Pediatric Public Health Initiative, 200 E 1st St, Flint, MI 48502, USA; 2 Division of Public Health, Michigan State University–Hurley Children’s Hospital Pediatric Public Health Initiative, 200 E 1st St, Flint, MI 48502, USA; jlachan1@hurleymc.com (J.L.); cejatiff@msu.edu (T.C.); 3 Department of Pediatrics and Human Development, Division of Public Health, Michigan State University–Hurley Children’s Hospital Pediatric Public Health Initiative, 200 E 1st St, Flint, MI 48502, USA; hannamon@msu.edu

**Keywords:** fruit and vegetable prescriptions, food security, food access, dietary patterns, children, primary prevention, low-income, pediatric, fruits and vegetables

## Abstract

Though fruit and vegetable consumption is essential for disease prevention and health maintenance, intake among children fails to meet dietary recommendations. Limited access to and the affordability of fresh produce, particularly among low-income youth, are barriers to adequate intake. To address these challenges, researchers and pediatricians in Flint, Michigan, expanded a successful fruit and vegetable prescription program that provides one $15 prescription for fresh fruits and vegetables to every child at every office visit. Vendors include the downtown farmers’ market and a local mobile market. This study describes baseline characteristics, dietary patterns, food access, and food security among 261 caregiver–child dyads enrolled August 2018–March 2019. The child-reported mean daily intake of vegetables (0.72 cups ± 0.77), dairy products (1.33 cups ± 1.22), and whole grains (0.51 ounces ± 0.49) were well below recommendations. Furthermore, 53% of children and 49% of caregivers who completed the food security module indicated low or very low food security. However, there were no statistically significant differences in the child consumption of fruits and vegetables between households that reported high versus low food security (*p* > 0.05). Results validate and raise deep concerns about poor dietary patterns and food insecurity issues facing Flint children, many of whom continue to battle with an ongoing drinking water crisis. Additional poverty-mitigating efforts, such as fruit and vegetable prescription programs, are necessary to address these gaps.

## 1. Introduction

Though fruit and vegetable consumption is essential for proper growth and development [1,2,3,4], as well as for the prevention of chronic disease [5,6,7,8,9], intake among children fails to meet dietary recommendations [10,11,12,13]. While small improvements in total fruit intake among children have been demonstrated in recent years, vegetable consumption remains very low among children of all socio-demographic groups. The most recent available data suggest that only children ages 2–5 are meeting Healthy People 2020 targets for fruits, and no socio-demographic groups of children are meeting targets for vegetable intake [14]. Limited access to and the affordability of fresh produce, particularly among low-income youth, are important barriers to adequate consumption [15,16,17]. To address these challenges, researchers partnered with pediatricians in Flint, Michigan, USA, to expand a successful fruit and vegetable prescription program that provides one $15 prescription (voucher) for fresh fruits and vegetables to every child at each office visit. Participating vendors include the downtown farmers’ market and a local mobile market, which accepts orders for fruit and vegetable boxes delivered to locations throughout the city.

Primary care providers serve as the leading source of health information among patients of all ages [18,19,20,21]. As a result, providers have the opportunity to educate individuals about the importance of proper nutrition and the connection between food and health [22,23]. Pediatricians, who often follow children from infancy to young adulthood, are uniquely positioned to influence lifelong dietary behaviors [24,25,26]. With mounting evidence that higher fruit and vegetable consumption during childhood is associated with reductions in chronic diseases in adulthood [5,24,27,28], both the immediate and long-term implications of fruit and vegetable prescription programs for pediatric patients could be substantial.

Unlike recent programs that have focused on fruit and vegetable prescriptions as a disease-management approach for adults with diet-related health conditions [18,29,30,31,32], the current project is a primary prevention program that emphasizes the critical role of fruits and vegetables in the prevention of chronic disease during formative childhood years [33]. To date, only two studies have examined fruit and vegetable prescription programs focused on primary prevention during childhood [15,34]. A previous qualitative study with caregivers of pediatric patients who received prescriptions for fresh fruits and vegetables from their pediatrician reported improvements in household food security, access to fresh foods, and child dietary patterns [15]. Similarly, evidence has suggested that pediatric fruit and vegetable prescriptions are an effective method to address food insecurity among low-income families [34]. The goal of the one-year pediatric fruit and vegetable prescription program study is to measure the influence of prescriptions on the dietary patterns of children, particularly changes in daily servings of fruits and vegetables, as well as household food security. Researchers hypothesize that children who participate in the fruit and vegetable prescription program will have improved dietary patterns and food security over 12 months. The purpose of the current study was to explore baseline characteristics, food security, food access, and dietary patterns among patients at a pediatric clinic in a low-income urban area that introduced a fruit and vegetable prescription program. Because the city continues to experience a lead-in-water public health crisis [35] which is aggravated by poor nutrition [36,37,38,39], researchers further examined relationships between food security and child dietary patterns among children who live in Flint.

## 2. Materials and Methods

### 2.1. Study Population

Nearly 60% of children who reside in Flint, Michigan, live below the poverty line [40], and the community struggles with a limited number of full-service grocery stores operating within the city [41]. These persistent challenges with food access and affordability have been further exacerbated by a well-publicized lead-in-water public health crisis [35,42]. In August 2015, a large university-affiliated residency training pediatric clinic relocated to the second floor of a downtown farmers’ market and established a pediatric fruit and vegetable prescription program [15]. In August 2018, this successful program was expanded to a second clinic in Flint to test its replicability in a pediatric office outside of a farmers’ market building. The second clinic is located several miles from the downtown farmers’ market and has approximately 3000 pediatric patients, most of whom live in Flint and receive public health insurance. The current study is an examination of the pediatric prescription program at the second clinic, located away from the downtown farmers’ market.

### 2.2. Study Design

This was a cross-sectional analysis of self-reported data from a convenience sample of caregiver–child dyads at their pediatrician’s office. Data were collected from caregiver–child dyads immediately prior to receiving a $15 prescription for fresh fruits and vegetables.

### 2.3. Pediatric Fruit and Vegetable Prescription Program

Similar to medical prescriptions, pediatric fruit and vegetable prescriptions are written by physicians and given to patients. In the current program, every patient receives a $15 fruit and vegetable prescription at each clinic visit to be redeemed for fresh produce at the Flint Farmers’ Market or Flint Fresh (a mobile market that offers free delivery of fresh produce boxes throughout Flint). Vendors ensure that prescriptions are treated as gift certificates or vouchers that may be redeemed exclusively for fresh fruits and vegetables. Prescriptions are printed with an expiration date 90 days from distribution. Unlike previous prescription programs that required physicians to identify at-risk patients through diet-related health conditions or income level, the fruit and vegetable prescriptions in the current program are designed to highlight the particular importance of healthy dietary patterns in the prevention of disease. Prescriptions are written by pediatricians and given to all patients, regardless of medical condition or household income, at the conclusion of their office visits.

### 2.4. Participants and Data Collection

After approval from the Michigan State University Institutional Review Board (Study00000666, titled “Fruit and Vegetable Prescription Program”), beginning in August 2018, all patients (0–18 years) at the partnering pediatric clinic received a $15 prescription for fresh fruits and vegetables during each office visit to be redeemed for fresh produce at the farmers’ market or local mobile market. Caregivers whose children were between the ages of 8 and 18 years and spoke English were invited to participate in the research study. To ensure collection of valid and reliable dietary data from children, researchers restricted enrollment in the study to children ages 8–18 years. Following caregiver consent and child assent, caregiver–child dyads (one caregiver and one child from each family) separately completed a series of demographic questions as well as survey questions to assess dietary patterns, food security, and food access. All data were collected using a digital platform (Michigan State University Qualtrics). This study was conducted in accordance with the Declaration of Helsinki.

### 2.5. Evaluation Tools

#### 2.5.1. Food Security

Caregivers completed the US Household Food Security Module: Six-Item Short Form, developed by the National Center for Health Statistics, to measure household food security and hunger [43]. The sum of affirmative responses (“often,” “sometimes,” “yes,” “almost every month,” and “some months but not every month”) to the six questions served as the household’s raw score. A food security status was assigned based on this calculated raw score (0–1 = high/marginal food security; 2–4 = low food security; and 5–6 = very low food security). A sample item was, “In the last 12 months, were you ever hungry but didn’t eat because there wasn’t enough money for food?” For the current analysis, a dichotomous summary score was created (0–1 = high/marginal food security; 2–6 = low/very low food security).

Children ages 12 years and older completed the Self-Administered Food Security Survey Module for Youth [44]. Though the internal validity of the module was found adequate for children ages 12 years and older, its use is not recommended for younger children. Therefore, the tool was used only with children 12 years of age and older. The sum of affirmative responses (“a lot” or “sometimes”) to the nine questions in the Child Food Security Module is the respondent’s raw score on the scale. A food security status was assigned by raw score (0–1 = high/marginal food security; 2–5 = low food security; and 6–9 = very low food security). A sample item was, “Did the food that your family bought run out, and you didn’t have money to get more?” For the current analysis, a dichotomous summary score was created (0–1 = high/marginal food security; 2–9 = low/very low food security).

#### 2.5.2. Dietary Patterns

The dietary patterns in children (8–18 years of age) were measured using the Block Kids Food Screener, which has previously demonstrated good relative validity for children and adolescents [45]. The 41-item Block Kids Food Screener, which assessed the frequency and quantity of foods and beverages consumed during the previous week, was self-administered. A research assistant was available to help younger children (under the age of 10 years) when necessary.

#### 2.5.3. Food Access

Caregivers completed selected questions from the Food Attitudes and Behaviors Survey (FAB) [46] to specifically examine access to fruits and vegetables. Responses were answered on a 5-point Likert scale (1 = “strongly disagree” to 5 = “strongly agree”). For analysis, “strongly agree” and “agree” were combined into “agree,” while “strongly disagree” and “disagree” were combined into “disagree.” A sample item was, “It’s hard for me to purchase fruits and vegetables in my neighborhood.” Caregivers also completed 4 questions from the Michigan Behavioral Risk Factor Surveillance Survey (MBRFSS) related to fruit and vegetable quality and access in neighborhood stores. Responses were answered on a 5-point Likert scale (1 = “always” to 5 = “never”). For analysis, “always” and “usually” were combined into “usually,” while “rarely” and “never” were combined into “rarely.” A sample item was “How often is a variety of good-quality orange-colored vegetables, such as sweet potatoes, pumpkin, winter squash, or carrots, available at this location?”

## 3. Results

A total of 261 caregiver–child dyads from Flint enrolled in the study between August 2018 and March 2019 (Table 1). Most of the caregivers (mean age of 40.0 ± 10.4 years) were African American (77.4%) and female (89.3%). Similarly, most of the children (mean age of 12.8 ± 2.9 years) were African American (79.7%) and female (54.0%). The majority of caregivers who completed the survey reported participation in the Supplemental Nutrition Assistance Program (SNAP) (56.3%, 147/261). Fifty-eight percent (152/261) reported that their child(ren) received free or reduced-price school meals. Seventy-six percent (198/261) of caregivers reported participation in either or both SNAP and free or reduced-price school meals.

### 3.1. Food Security

Approximately half of the caregivers who completed the US Household Food Security Module (48.7%, *n* = 127) reported low food security (29.9%, *n* = 78) or very low food security (18.8%, *n* = 49). A total of 164 children, 12 years of age and older, completed the Self-Administered Food Security Survey Module for Youth. Over half of these children (53.0%, *n* = 87) reported low food security (41.5%, *n* = 68) or very low food security (11.6%, *n* = 19). There was a low correlation between caregiver and child food security categories (r = 0.13, *p* = 0.10). Though child-reported food security scores were slightly lower for children who completed the questionnaire during summer months (when school was not in session) than for children who completed the survey during the academic year, the difference was not statistically significant (*p* = 0.472). Regardless of the time of year, over half of the children who completed the food security survey reported low or very low food security. Specifically, 58.3% (21/36) of the children reported low food security (44.4%, *n* = 16) or very low food security (13.9%, *n* = 5) during the summer, compared to 51.6% (66/128) of children who reported low food security (40.6%, *n* = 52) or very low food security (10.9%, *n* = 14) during the school year.

### 3.2. Dietary Patterns

The dietary patterns were analyzed using Block Kids Food Screener reports of vegetables (excluding potatoes and legumes), fruits (with juices), whole fruits, dairy, whole grains, fiber, and added sugars (Table 2). The reported intake failed to meet current dietary recommendations for the majority of children who completed the screener. The mean intake of vegetables was 0.72 ± 0.77 cups per day, with 93.3% reporting a daily consumption of fewer than 2 cups. The average daily consumption of fruits (with juices) was 1.51 ± 1.30 cups, with 58.7% of children reporting less than 1.5 cups of fruit per day, and 73.4% reporting fewer than 2 cups. The mean intake of whole fruit was 0.73 ± 0.83 cups per day, with 87.3% reporting a daily consumption of less than 1.5 cups per day and 92.5% reporting fewer than 2 cups per day. The mean intake of dairy products was 1.33 ± 1.12 cups per day, with 92.9% of children reporting less than 3 cups of dairy foods per day. The mean intake of protein sources was 3.49 ± 4.39 ounces per day, with 84.1% of children reporting less than 5 ounces of protein per day. The mean daily intake of whole grains was 0.51 ± 0.49 ounces, with 100% of children reporting a consumption of fewer than 3 ounces of whole grains per day. The mean daily fiber intake was 11.2 ± 9.1 g, with 94.8% of the children reporting daily intakes below 26 g of fiber. The mean intake of added sugars was 9.6 ± 8.4 g, with 35.3% of the children reporting daily intakes of more than 10 g of added sugar, and 7.1% reporting daily intake of at least 25 g of sugar.

### 3.3. Food Access

Considering the ongoing challenges with the lead-in-water crisis in Flint, caregiver-reported food access measures were collected. Caregivers were asked whether they agreed or disagreed with the following statement: “I do not have safe water to wash and prepare fruits and vegetables.” Over half of the caregivers disagreed (65.7%, 165/251). Nearly half (49.0%, 124/253) disagreed that fruits and vegetables cost too much, and the majority of caregivers (70.1%, 176/251) disagreed that it was hard to purchase fruits and vegetables in their neighborhood. Approximately 40% (41.5%, 105/253) agreed to the statement, “When I eat out, it’s easy to get fruits and vegetables.” When specifically asked about fast food, however, only 14.2% (37/259) agreed with the statement, “Fast food places offer enough choices of fruits and vegetables on their menus.”

Additional questions from the MBRFSS focused on fruit and vegetable quality and access as impacted by the store. The majority of caregivers reported that distance to the store from home was rarely (25.7%, 67/261) or never (33.7%, 88/261) a problem. Over 80% (80.5%, 210/261) reported that there was always (57.5%, 150/261) or usually (23.0%, 60/261) a variety of high-quality dark green vegetables at the store. Similarly, 72.0% said that there was always (43.7%, 114/261) or usually (28.4%, 74/261) a variety of high-quality orange vegetables. Over 80% (82.4%, 215/261) reported that there was always (61.3%, 160/261) or usually (21.1%, 55/261) a variety of quality fruits.

### 3.4. Relationship between Fruit and Vegetable Intake and Food Security and Access

Using independent samples *t*-tests, researchers examined whether there was a difference in the mean daily servings of vegetables (excluding potatoes and legumes), fruits with juices, or whole fruits between those children who reported low or very low food security and those children who reported high or marginal food security. There were no statistically significant differences based on food security groups for the fruit and vegetable dietary items (*p* > 0.05). Additionally, researchers assessed differences in fruit and vegetable intake and food access in relation to water safety, cost, purchasing in the neighborhood, distance from the store, and variety of high-quality fruits and vegetables (Table 3). There were no statistically significant differences in the mean daily intake of vegetables, fruits with juices, or whole fruits in any of the measures.

## 4. Discussion

The current study is the first to examine baseline dietary patterns and food security among pediatric patients immediately prior to the introduction of a clinic-wide fruit and vegetable prescription program. Central to our findings were consistently poor dietary patterns among our sample of children. The majority failed to reach dietary recommendations, with less than 10% of the entire sample meeting daily recommendations for vegetables, whole grains, dairy, and fiber. This finding, which is consistent with previous literature focused on dietary patterns of children and adolescents living in low-income urban communities [10,47,48], validates and continues to raise deep concerns about poor dietary patterns among children in Flint, many of whom continue to battle an ongoing drinking water crisis that is worsened by hunger and poor nutrition [38,49,50]. Continued poverty-mitigating efforts, such as fruit and vegetable prescriptions, are necessary to address pervasive dietary concerns among children in Flint.

Additionally, over half of the children who participated in the study reported low or very low food security. Though not statistically significant, child-reported food security was higher during the school year as compared to summer months. This apparent lack of reliable access to sufficient quality and quantity of food for children living in Flint is extremely concerning given evidence that food insecurity in children is associated with numerous negative health outcomes, including childhood overweight and obesity [51,52,53,54]. In 2015, the American Academy of Pediatrics recommended that providers screen all households with children for food insecurity [55]. However, many providers do not screen, indicating a lack of resources to support those families who screen positive [56]. Pediatric fruit and vegetable prescriptions could be replicated in primary care practices across the country to actively address food insecurity in children. Additionally, given strong evidence that fruit and vegetable intake is consistently and positively associated with income [11,26,57], fruit and vegetable prescription programs are likely to disproportionately benefit children and adolescents suffering from food insecurity.

In sharp contrast to previous research demonstrating that residents of food desert communities, such as Flint, are frequently challenged with limited access to fresh, high-quality foods [58,59], caregivers in the current study reported very few barriers to accessing fresh, high-quality fruits and vegetables. Though low-income neighborhoods often have poorer quality and fewer healthy food options than higher-income neighborhoods [60,61], few caregivers in the current study revealed challenges with food access within their neighborhoods. Furthermore, when assessing the relationship between child-reported fruit and vegetable intake and food access, there were no significant associations between the mean daily intake of vegetables (excluding potatoes and legumes), fruits (with juices), or whole fruits and various measures of food access. It is important to note, however, that this lack of association could be explained by extremely poor intake of fruits and vegetables across our entire sample of children, and our study was not powered to detect small differences between the groups. Unlike other food access questions, the majority of caregivers did indicate that local fast food restaurants offered few fruit and vegetable options. It has been suggested that Americans are increasingly eating meals away from home, a behavior that has consistently been associated with adverse nutritional consequences [62,63,64]. This finding is noteworthy because fast food restaurants have become increasingly popular in recent decades [65], and the availability of high-nutrient foods, such as fruits and vegetables, is often inadequate.

Limitations of the current study should be acknowledged. Our sample was specific to one low-income urban city. As such, results may not be generalizable to other communities. Additionally, there may have been selection bias, as responses from caregivers and patients who chose not to complete the survey may have differed from those who voluntarily agreed to complete the survey. However, the characteristics of the study population closely match those of the source patient population at the pediatric clinic, which consists primarily of low-income, minority patients receiving public health insurance. Finally, the cross-sectional study design did not allow researchers to investigate the impact of the prescription program over time, but this was an important preliminary study to examine baseline dietary patterns and food insecurity among children newly exposed to a fruit and vegetable prescription program.

## 5. Conclusions

Unlike the majority of fruit and vegetable prescription programs that primarily target income-eligible adults with nutrition-related health conditions [22,29,30,66], the current program focused on preventing disease and promoting healthy eating patterns through improved affordability and exposure to fresh, high-quality produce. Pediatricians and primary care providers are in the unique and favorable position to help children develop healthy eating behaviors that are likely to continue throughout their lives [33]. Providing a fruit and vegetable prescription to every child demonstrates physicians’ recognition of the important role of fresh, high-nutrient foods in health promotion and prevention of chronic disease. Future research in Flint will investigate caregiver- and child-reported changes in food security scores, dietary patterns, food access, and clinical markers in relation to participation in the fruit and vegetable prescription program. Additionally, future research will explore the impact of other food assistance programs on measures of dietary intake, reported food security, and food access.

## Figures and Tables

**Table 1:** Demographics of caregivers and children enrolled in study.

Demographics	Caregivers (*n* = 261)	Children (*n* = 261)
Mean age	40.0 ± 10.4 years	12.8 ± 2.9 years
Race		
African American	77.4%	79.7%
White	15.3%	14.2%
Other/Not reported	7.3%	6.1%
Sex		
Female	89.3%	54.0%
Male	10.7%	46.0%
Education		
High school degree or less	33.3%	
Some college/Technical school/Associate’s degree	39.8%	
Bachelor’s degree	14.6%	
Graduate degree	8.0%	
Other/Not reported	4.3%	

**Table 2:** Child-reported dietary patterns.

Foods and Beverages	Dietary Recommendations (9–18 Years of Age) *	Percent Meeting Current Dietary Recommendations
Vegetables	2–3 cups per day	6.7%
Fruits with juices	1.5–2 cups per day	41.3%
Whole fruits	n/a	n/a
Dairy	3 cups per day	7.1%
Protein sources	5–6.5 ounces per day	15.9%
Whole grains	3–4 ounces per day	0.0%
Fiber	26–38 g per day	5.2%
Added sugar	<25 g per day	92.9%

* Dietary recommendations for vegetables, fruits, dairy, protein sources, and whole grains were based on the United States Department of Agriculture (USDA) MyPlate. Dietary recommendations for fiber and added sugar were based on the American Heart Association guidelines for children.

**Table 3:** Associations between child-reported fruit and vegetable intake and caregiver-reported food access.

Prompt for Caregiver-Reported Food Access	Child-Reported Intake	Preferred Response Group (Disagree/Strongly Disagree), Mean Intake	Non-Preferred Response Group (Strongly Agree/Agree/Neutral), Mean Intake	*p*-Value
It is hard for me to eat more fruits and vegetables because I do not have safe water to wash and prepare them.	Vegetables	0.68 ± 0.78	0.76 ± 0.74	0.45
	Fruits with juices	1.54 ± 1.36	1.49 ± 1.27	0.78
	Whole fruits	0.73 ± 0.83	0.64 ± 0.68	0.36
They cost too much.	Vegetables	0.76 ± 0.73	0.70 ± 0.82	0.54
	Fruits with juices	1.63 ± 1.34	1.42 ± 1.29	0.23
	Whole fruits	0.71 ± 0.77	0.69 ± 0.79	0.86
It is hard for me to purchase fruits and vegetables in my neighborhood.	Vegetables	0.75 ± 0.75	0.70 ± 0.84	0.63
	Fruits with juices	1.47 ± 1.27	1.65 ± 1.43	0.32
	Whole fruits	0.68 ± 0.78	0.73 ± 0.75	0.67
		**Preferred Response Group (Rarely/Never), Mean Intake**	**Non-Preferred Response Group (Always/Usually/Sometimes), Mean Intake**	***p*-Value**
How often does the distance from your home to a full service grocery store make it difficult for you to buy the variety and quality of fresh fruits and vegetables you would like?	Vegetables	0.67 ± 0.71	0.80 ± 0.85	0.22
	Fruits with juices	1.49 ± 1.29	1.55 ± 1.32	0.75
	Whole fruits	0.73 ± 0.80	0.65 ± 0.73	0.42
		**Preferred Response Group (Always/ Usually), Mean Intake**	**Non-Preferred Response Group (Rarely/Never/ Sometimes), Mean Intake**	***p*-Value**
How often is a variety of good-quality dark green vegetables, such as broccoli, romaine, chard, collard greens, or spinach, available at this location?	Vegetables	0.70 ± 0.70	0.84 ± 1.00	0.35
	Fruits with juices	1.56 ± 1.26	1.34 ± 1.48	0.30
	Whole fruits	0.73 ± 0.76	0.55 ± 0.79	0.15
How often is a variety of good-quality orange-colored vegetables, such as sweet potatoes, pumpkin, winter squash, or carrots, available at this location?	Vegetables	0.72 ± 0.70	0.74 ± 0.94	0.81
	Fruits with juices	1.54 ± 1.25	1.46 ± 1.44	0.68
	Whole fruits	0.75 ± 0.77	0.56 ± 0.75	0.09
How often is a variety of fresh, frozen, or canned fruits available at this location?	Vegetables	0.70 ± 0.70	0.86 ± 1.04	0.21
	Fruits with juices	1.50 ± 1.20	1.60 ± 1.72	0.72
	Whole fruits	0.71 ± 0.73	0.62 ± 0.94	0.48
